# Ageing *Fxr* Deficient Mice Develop Increased Energy Expenditure, Improved Glucose Control and Liver Damage Resembling NASH

**DOI:** 10.1371/journal.pone.0064721

**Published:** 2013-05-20

**Authors:** Mikael Bjursell, Marianne Wedin, Therése Admyre, Majlis Hermansson, Gerhard Böttcher, Melker Göransson, Daniel Lindén, Krister Bamberg, Jan Oscarsson, Mohammad Bohlooly-Y

**Affiliations:** AstraZeneca R&D, Mölndal, Sweden; CIMA, University of Navarra, Spain

## Abstract

Nuclear receptor subfamily 1, group H, member 4 (Nr1h4, FXR) is a bile acid activated nuclear receptor mainly expressed in the liver, intestine, kidney and adrenal glands. Upon activation, the primary function is to suppress cholesterol 7 alpha-hydroxylase (Cyp7a1), the rate-limiting enzyme in the classic or neutral bile acid synthesis pathway. In the present study, a novel *Fxr* deficient mouse line was created and studied with respect to metabolism and liver function in ageing mice fed chow diet. The *Fxr* deficient mice were similar to wild type mice in terms of body weight, body composition, energy intake and expenditure as well as behaviours at a young age. However, from 15 weeks of age and onwards, the *Fxr* deficient mice had almost no body weight increase up to 39 weeks of age mainly because of lower body fat mass. The lower body weight gain was associated with increased energy expenditure that was not compensated by increased food intake. Fasting levels of glucose and insulin were lower and glucose tolerance was improved in old and lean *Fxr* deficient mice. However, the *Fxr* deficient mice displayed significantly increased liver weight, steatosis, hepatocyte ballooning degeneration and lobular inflammation together with elevated plasma levels of ALT, bilirubin and bile acids, findings compatible with non-alcoholic steatohepatitis (NASH) and cholestasis. In conclusion, ageing *Fxr* deficient mice display late onset leanness associated with elevated energy expenditure and improved glucose control but develop severe NASH-like liver pathology.

## Introduction

Nuclear receptors are ligand-activated transcription factors involved in a variety of physiological and developmental processes. Nuclear receptor subfamily 1, group H, member 4 (Nr1h4, Farnesoid X receptor (FXR)), is activated by bile acids and highly expressed in liver, kidney, adrenal glands and intestine [1], [2]. FXR is important for monitoring bile acid homeostasis and plays a central role in the regulation of lipid and glucose control [3]–[7]. Hepatic FXR is involved in a feedback inhibition loop regulating Cyp7a1 expression, where bile acid activated FXR up regulates the small heterodimer partner (SHP), an atypical nuclear receptor lacking a DNA binding domain. SHP suppresses Cyp7a1 expression by inhibiting liver receptor homologue 1 (Lrh-1), an obligate transcription factor for Cyp7a1 expression [8]. Intestinal FXR regulates bile acid synthesis via a pathway including up regulation of fibroblast growth factor (FGF) 15 in rodents and FGF19 in humans, which acts on liver FGF receptor 4 to suppress transcription of Cyp7a1 [9]–[11].

There are several previous reports describing effects of *Fxr* deficiency in mice. However, to our knowledge, only two *Fxr* deficient mouse lines have been described previously, one developed at Deltagen Inc. [12], [13], and one developed by Sinal *et.al.* [3]–[7], [9], [14]–[26]. The Deltagen derived *Fxr* deficient mouse line displays moderately elevated plasma bile salt levels, lower hepatic bile salt export pump (*Abcb11*) expression, slightly higher body and liver weight at 3 months of age together with lower fed and fasting blood glucose and changes in intestinal glucose absorption [12], [13]. The *Fxr* deficient mouse line originally derived from Sinal *et.al.* [5] has been more extensively studied and studies of this mouse model have shown the importance of *Fxr* for bile acid, lipid and glucose homeostasis, development of nephropathy, cancer and liver function in a variety of settings. Zhang and colleagues showed that *Fxr* deficient mice display unaltered fasting glucose but mild glucose intolerance and insulin resistance together with low hepatic levels of insulin stimulated IRS-2 phosphorylation [15]. Other studies on the *Fxr* deficient mouse line derived from Sinal *et.al.* reveal age dependent increase in fasting blood glucose, impaired glucose tolerance and attenuated peripheral glucose disposal as demonstrated by clamp studies [4], [7]. More recently, *Fxr* deficient mice bred on a leptin deficient genetic background displayed lower body weight and body fat mass together with lower blood glucose and insulin levels, improved glucose tolerance and insulin sensitivity, despite low energy expenditure [22]. In addition, *Fxr* deficient mice fed a high fat diet (HFD) also displayed lower body weight and plasma leptin levels together with low blood glucose, insulin and improved glucose tolerance [22] indicating that *Fxr* deficiency results in improved glucose control in obese animals. Also, *Fxr* deficiency bred on an *Ldlr* deficient background fed a HFD resulted in lower body weight at 3 months of age, dyslipidemia and elevated alanine transaminase (ALT) and alkaline phosphatase (ALP) levels. In addition, liver histopathology revealed mild macrosteatosis and focal inflammatory cell accumulation when *Fxr*/*Ldlr* deficient mice were fed chow diet, and severe macrosteatosis, hepatocyte ballooning, panlobular inflammation together with elevated hepatic expression of TNF-α and ICAM-1 when *Fxr*/*Ldlr* deficient mice were fed a HFD. The *Fxr*/*Ldlr* deficient mice were suggested to be a model displaying NASH [24]. FXR has also been reported to be important for liver regeneration and to prevent cell death [17], [18].

The present study aimed to further investigate the importance of a functional *Fxr* gene in the regulation of metabolism in a novel mouse line carrying a targeted deletion of the *Fxr* gene in order to evaluate FXR as a potential drug target. The aim of this study was to investigate the changes in energy metabolism in ageing *Fxr* deficient mice given ordinary low fat chow diet. In brief, we show that *Fxr* is of major importance for regulation of energy expenditure, glucose- and lipid metabolism and liver function in ageing mice even though they were not metabolically challenged.

## Materials and Methods

### Ethics Statement

All experiments were approved by Gothenburg Ethics Committee for Experimental Animals.

### Generation of *Fxr* null mice

**Figure 1:**
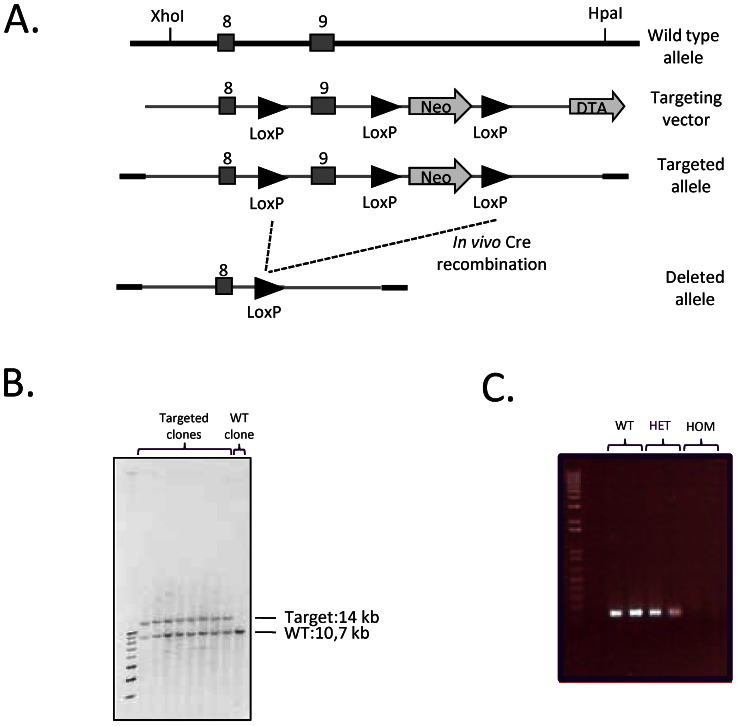
*Fxr* deficient mouse line generation. (A) Schematic diagram of the *Fxr* gene and targeting vector. (B) Southern blot analyses of 8 targeted ES cell clones (lane 1–8) and a wild type control clone (lane 9), using *HindIII* restriction enzyme, resulting in wild type fragments of 10649 bp and targeted fragments of 13975 bp. (C) RT-PCR analyses of liver biopsies from WT (lane 1–2), *Fxr* heterozygous knock out (lane 3–4) and *Fxr* homozygous knock out (lane 5–6) mice. The RT-PCR products represent presence (lane 1–4) or absence (lane 5–6) of exon 9 in the *Fxr* gene.

A Lox-P strategy was used to target the *Nr1h4* (*Fxr*) locus in order to generate *Fxr* deficient mice. In essence, a 14.2 kb C57BL/6 mouse genomic sub clone was used as targeting vector, containing a floxed neomycin phosphotransferase selection marker cassette after the coding sequence in the untranslated region (UTR) and a single Lox-P site inserted in intron 8 of the *Fxr* locus (illustrated schematically in Fig. 1A). After linearization by XhoI, the targeting construct was electroporated into embryonic stem (ES) cells (derived from C57/J, Genome Systems, St. Louis, USA) and neomycin-resistant clones were selected in G-418-containing (300 µg/ml) media. Thus, the *Fxr* deficient mouse line was established on a pure C57BL/6 genetic background. Targeted clones were identified by PCR screening over the short arm homology of the construct and further confirmed by southern blot analyses using *HindIII* restriction enzyme (Fig. 1B). One clone was expanded and injected into BALB/C blastocysts to generate chimeric mice. Chimeric males were crossed to C57BL/6N female mice (Charles River, Sulzfeld, Germany) and genotyping of the black coat colour offspring was performed. The following primers were used to identify offspring with the targeted allele: forward neo-specific primer (F) 5′-GCTGACCGCTTCCTCGTGCTTT-3′ together with reverse primer within the short arm homology (R1) 5′-GGGAGAAGACTGCCACAGTTGA -3′. Heterozygous mice carrying Lox-P DNA sites were bred to ROSA26Cre mice [27] AstraZeneca in-house backcrossed more than 20 generation towards C57BL/6N (Charles River) in order to generate heterozygous *Fxr* deficient mice. The mice were identified using the following genotyping primers: intron specific: 5′-CCAGTGACCCGTGCCTGTAATGT -3′; intron specific; 5′- GGGAGAAGACTGCCACAGTTGA -3′ (627 bp products for the recombined allele and 2 253 bp products for the wild-type allele). Heterozygous *Fxr* deficient mice were intercrossed to generate homozygous *Fxr* animals, which proved to be viable.

To verify an *Fxr* null mutation, total RNA was prepared from livers of 12 weeks old homozygous, heterozygous, and wild-type littermates using the RNA STAT-60 Kit according to the manufacturer' instructions (Tel-Test Inc, Friendswood, USA). cDNA was synthesized using Superscript TM II RNAse H - Reverse Transcriptase and random hexamer primers (Life Technologies, Frederick, USA). RT-PCR was performed using the following primers: forward: Ex 5′-CTTGATGTGCTACAAAAGCTGTG-3′; reverse: Ex 5′-ACTCTCCAAGACATCAGCATCTC-3′ (Fig. 1C) to show exon 9 deletion in the *Fxr* deficient mice.

The *Fxr* heterozygous mouse colony was expanded by breeding to C57BL/6N (Charles River, Sulzfeld, Germany) and heterozygous intercross was performed to produce experimental (*Fxr* KO) and wild type (WT) littermate control cohorts, having a pure C57BL/6N genetic background. All experiments were performed using the same individuals of male mice over the course of the study.

Male *Fxr* KO and WT littermates were housed individually in a temperature controlled room (22°C) with a 12 hours light-dark cycle. They had access to normal chow diet (R36, Lactamin AB, Stockholm, Sweden) and water *ad libitum*. The R36 chow diet contained (weight%): 3.5% cellulose, (energy%): 22.9% protein, 67.1% carbohydrate and 9.6% fat. The main sources of proteins were from soy, grain and potatoes. Carbohydrate source was mainly grains and main fat source was soy. The energy density of R36 was 3.08 kcal/g.

The mice were initially studied in terms of gross appearance, cage side behaviours and basic responses, including assessment of balance, sensory pain, acoustic startle and grip strength (Grip strength meter, Columbus Instruments, Columbus, USA) as described previously [28]. To assess potential differences in behaviour in the *Fxr* KO mice, zero maze analysis was performed to investigate anxiety-like behaviours, passive avoidance to investigate memory and learning ability and forced swim test to study depression as described [28]. Open field locomotor behaviours were investigated during day time in specifically designed activity boxes (Kungsbacka mät-och reglerteknik, Kungsbacka, Sweden) as previously described [28].

### Body weight, indirect calorimetry, locomotor activity and food intake

Body weight of the *Fxr* KO mice (*n* = 8) and WT mice (*n* = 8) were recorded on a weekly basis from 4 weeks of age up to 10 weeks of age and then biweekly from 15 to 30 weeks of age and finally at 39 weeks of age. Body length (nose to base of the tail) was assessed at, 3, 7, 9 and 30 weeks of age. Assessment of indirect calorimetry, food and water consumption and locomotor activity was performed in a CLAMS system (Columbus Instruments, Columbus, USA) at thermoneutral temperature (set for WT mice to be 29.5°C) as previously described [28]. The mice were placed in the CLAMS calorimeter chambers with *ad libitum* access to diet and water for 72 hours.

In older mice, food intake was analysed over 48 hours in food deprived mice (12 hours) as previously described [29] with a minor modification: no initial incubation (80°C for 1 hour) of the cages was done. Total faeces produced over the measurement periods were collected and energy content of the faeces was determined with a bomb calorimeter (C 5000, IKA® Werke GmbH & Co. KG, Germany) according to manufacturers protocol.

### Body temperature and body composition

Rectal core body temperatures were recorded in conscious non-anaesthetised mice at day time (10.00–11.00 am) using a rectal probe [28]. Body composition was assessed by dual energy X-ray absorptiometry (DEXA, GE Lunar, Madison, USA) in isoflurane anaesthetised mice as previously described [28].

### Oral glucose tolerance test (OGTT), blood- and tissue sampling

OGTT was performed in 30 weeks old *Fxr* KO and WT mice as previously described [29]. Homeostasis model assessment (HOMA) was calculated as [fasting blood glucose (mM) *fasting blood insulin (ng/ml)/22.5].

The mice were terminated at 39 weeks of age. Before sacrifice, the mice were fasted for four hours before measuring blood glucose levels (Accu-check device, Roche Diagnostics, Mannheim, Germany). The mice were then anaesthetised by isoflurane inhalation, euthanised by cardiac puncture and blood was collected in EDTA coated tubes by cardiac puncture. Blood plasma was separated by centrifugation (2500 rpm, 10 min. 4°C) and snap frozen in liquid nitrogen. The following organs and tissues were dissected and weighed: brain, caecum, liver, epididymal white adipose tissue (WAT), retroperitoneal WAT, interscapular brown adipose tissue (BAT), kidneys, spleen, testis, heart and gall bladder. Liver tissue samples were taken for hepatic triglyceride content analysis. Tissue samples of liver, epididymal WAT, interscapular BAT and skin were processed for histological analysis by immersion fixation in 4% buffered formaldehyde solution for 24–48 hours, dehydrated in graded series of alcohol and embedded in paraffin before sectioning.

### Plasma analysis

Plasma levels of non-esterified fatty acids (NEFA), cholesterol, triglyceride, alanine aminotransferase (ALT), high density lipoprotein (HDL) and non-HDL cholesterol were determined as previously described [30]. Plasma levels of total bilirubin were measured using a colorimetric method (Kit No 11552414 216; BIL-T, Roche Diagnostics GmbH, Germany) and total bile acids were assessed by using an enzymatic method (Kit No Bl 3863, Randox Laboratoires Ltd, United Kingdom).

### Expression levels analysis

RNA extraction, cDNA synthesis and quantification by Taqman real time PCR was performed as previously described [31]. Primer and probe sequences are presented in Table 1. Taqman assays for Cyp8b1 and Abcb11 were purchased as assay on demand (Applied Biosystems, Foster City, CA).

**Table 1:** Sequences of primers & probes.

Gene	Forward sequence	Reverse sequence	Probe sequence
Ppara	Cacgatgctgtcctccttga	gtgtgataaagccattgccgt	Acaaagacgggatgctgatcgcg
Acox1	tgttgtccctatccgtgagattg	ggccgatatccccaacagt	Acccacaagcctctgccaggca
Acadm	Tgacggagcagccaatga	atggccgccacatcaga	Tgcttactgtgtgacagagccctccg
Cyp7a1	cattacagagtgctggccaaga	gatgctatctagtactggcaggttgt	ctcagctctggagggaatgccatttacttg
Cyp8b1	Mm00501637_s1		
Abcb11	Mm00445168_m1		
Srebp-1c	Cacggagccatggattgc	cccgggaagtcactgtcttg	catttgaagacatgcatccagctcatcaaca
Fasn	Cctggactcgctcatgggt	atttcctgaagtttccgcagc	Cgtcagatcctggaacgagaacacga
Scd1	Cctgcggatcttccttatcatt	gatctcgggcccattcg	Accatggcgttccagaatgacgtgt
Hmox1	tcaggtgtccagagaaggcttt	tcttccagggccgtgtagat	Agctggtgatggcttccttgta
Tnfa	Atggcccagaccctcaca	ttgctacgacgtgggctaca	tcagatcatcttctcaaaattcgagtgacaagc
Itgax	ccactgtctgccttcatattcatg	catggtctagagccaggtcaaag	ccaagacccaactaggtgacctccgaagta
Ccl5	Gcaagtgctccaatcttgca	cttctctgggttggcacaca	Tcgtgtttgtcactcgaaggaaccgc
Tlr4	tctgatcatggcactgttcttctc	tctgatccatgcattggtaggt	ccaggaagcttgaatccctgcatagaggtagt

Ppara; peroxisome proliferator activated receptor alpha;

Acox1; acyl-Coenzyme A oxidase 1, palmitoyl;

Acadm; acyl-Coenzyme A dehydrogenase, medium chain;

Cyp7a1; cytochrome P450, family 7, subfamily a, polypeptide 1;

Cyp8b1; cytochrome P450, family 8, subfamily b, polypeptide 1;

Abcb11; ATP-binding cassette, sub-family B (MDR/TAP), member 11;

Srepb1c; sterol regulatory element binding protein 1c;

Fasn; fatty acid synthase;

Scd1; stearoyl CoA desaturase 1;

Hmox1; heme oxygenase 1;

Tnfa; tumor necrosis factor alpha;

Itgax; cd11c or Itgax integrin alpha x;

Ccl5; rantes;

Tlr4; toll-like receptor 4.

### Histology

Epididymal WAT cell profile size, macrophage staining and quantification and interscapular BAT cell profile size and density was assessed in histological sections as previously described [30]. A histopathological examination and evaluation of epidermis and liver tissue samples was performed on routine hematoxylin- eosin stained sections and the degree of steatosis and inflammation was scored on a semi-quantitative 5 grade scale.

### Statistical analysis

All values are given as group mean ± SEM. Comparison between two groups was done by Student' T-test. Parameters over time, e.g. energy expenditure and RER, were analysed by a mixed model 2-way ANOVA using the SPSS software. Values of p<0.05 were considered significant. The data were log normalised when appropriate.

## Results

### Animals

*Fxr* deficient mice (*Fxr* KO) were generated by targeted deletion of a 3326 bp DNA segment of the coding region of exon 9 (Fig. 1A) and positive ES cell clones identified by southern blot (Fig. 1B). To confirm absence of *Fxr* transcript in the *Fxr* KO mice, RT-PCR analysis of mRNA prepared from liver biopsies were performed. As expected, no expression of *Fxr* was observed in the *Fxr* KO mice (Fig.1C). Intercross of heterozygous *Fxr* mice resulted in offspring of normal litter sizes. Of the male offspring; 22% (n = 12) were homozygous, 58% (n = 32) were heterozygous and 20% (n = 11) were wild type mice. All experiments were performed on the same individuals of single housed male mice.

The mice were analysed in a battery of tests to assess appearance, cage side behaviours, basic responses and functions, anxiety, memory and learning and depression [28] between 4–11 weeks of age. No significant differences were observed between *Fxr* KO mice and WT mice with respect to any parameter investigated (data not shown) indicating that *Fxr* deficiency does not alter gross appearance or behaviour in mice. Locomotor behaviours were investigated in more detail in an open field locomotor activity setup. No significant differences in day-time locomotor behaviours were observed at 9 weeks of age between *Fxr* KO mice and WT mice.

### Body weight and body composition

**Figure 2:**
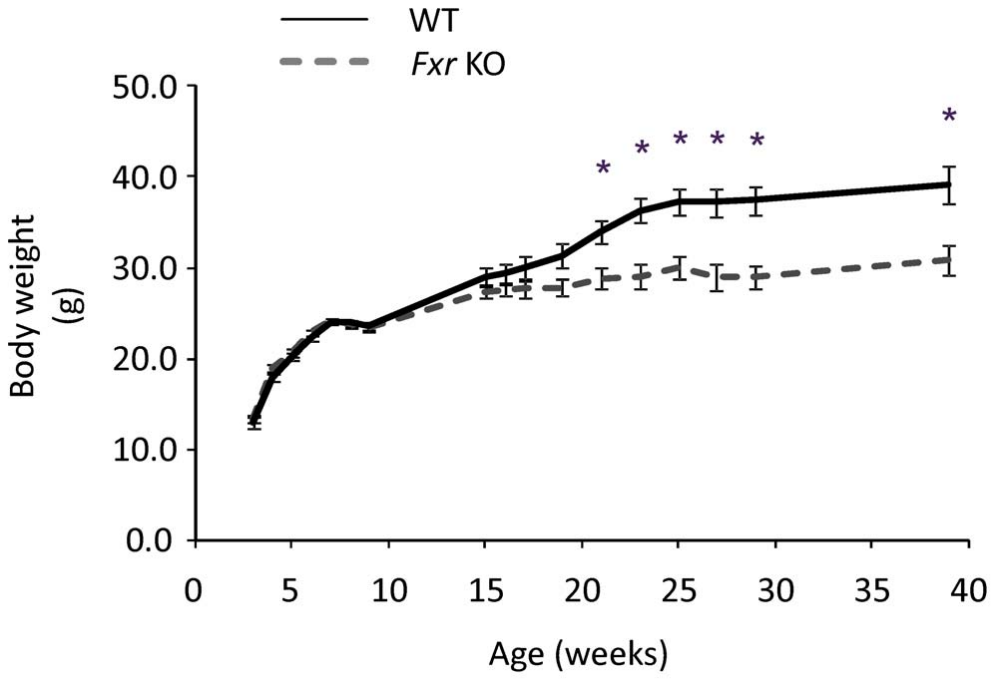
Body weight development. Body weight development over 39 weeks in WT (*n* = 8, black solid line) and *Fxr* KO mice (*n* = 8, grey dashed line). Statistical analysis was done by a repeated Student' T-test. * p<0.05 *Fxr* KO vs. WT mice.

Body weight did not differ significantly between young *Fxr* KO and WT mice. From 21 weeks of age and onwards, the *Fxr* KO mice displayed significantly lower body weight compared to the WT control mice (Fig. 2). At termination (39 weeks of age), the *Fxr* KO mice displayed approximately 8 g lower body weight compared to the WT mice (WT: 39.2±2.1 g; *Fxr* KO: 30.9±1.7 g;−21%; *p*<0.01). Body length was not significantly different at any time point assessed between *Fxr* KO and WT mice (data not shown).

Body composition was assessed at 9 weeks of age and then again at 30 weeks of age. At 9 weeks of age, no significant differences were observed in body lean mass, body fat mass or bone mineral content (BMC) between *Fxr* KO and WT mice (data not shown). However, bone mineral density (BMD) was slightly but significantly lower in the *Fxr* KO compared to WT mice (WT: 46.5±0.4 g/cm^2^; *Fxr* KO: 45.1±0.3 g/cm^2^, *p*<0.05). At 30 weeks of age, the *Fxr* KO mice displayed significantly lower BMD, BMC and body fat mass, whereas body lean mass was not significantly different between *Fxr* KO and WT mice (Table 2).

**Table 2:** Body composition at 30 weeks of age.

Parameter	WT	*Fxr* KO
Body fat mass (g)	10.93±1.24	5.01±0.66***
Relative body fat mass (% of bw)	31.14±2.31	18.18±1.62***
Body lean mass (g)	23.55±0.51	21.93±0.82
Relative body lean mass (g/cm bl)	2.24±0.04	2.08±0.07
Bone mineral content (g)	0.52±0.02	0.47±0.02*
Relative bone mineral content (mg/cm bl)	49.67±1.58	44.48±0.82*
Bone mineral density (mg/cm^2^)	51.78±0.71	48.40±1.00*

Body composition assessed by DEXA. Values are presented as group mean ± SEM. Body weight at assessment for WT mice (n = 8): 36.1±1.5 g, *Fxr* KO mice (n = 8): 28.2±1.4 g. Statistical analysis performed by Student's t-test.

* p<0.05;

*** p<0.001 *Fxr* KO vs. WT mice.

bw; body weight, bl; body length.

### Energy intake, energy expenditure, locomotor activity and core body temperature

The mice were analysed with respect to energy intake, faecal energy output, energy expenditure, core body temperature and locomotor activity initially at a younger age and then again at an about 30 weeks of age.

At 8 weeks of age, no significant differences were observed in terms of energy intake, faecal energy density, total faecal energy, energy uptake (energy intake minus faecal energy) or water intake between the *Fxr* KO and WT mice (data not shown). No significant difference was observed in respiratory exchange ratio (RER), energy expenditure, core body temperature or spontaneous locomotor or rearing activity between *Fxr* KO and WT mice at that age (data not shown).

The mice were reanalyzed at 29–31 weeks of age. Total energy intake was not significantly different, whereas relative energy intake (relative to body weight) was significantly increased in the *Fxr* KO mice compared to WT mice (Table 3). Total water intake was not significantly different, whereas relative water intake (relative to body weight) was significantly higher in the *Fxr* KO compared to the WT mice (Table 3). Total faeces production, faeces energy density, total faecal energy output or energy uptake (energy intake minus energy lost in faeces) were not significantly different between *Fxr* KO and WT mice, whereas relative energy uptake (relative to body weight) was significantly increased in the *Fxr* KO compared to the WT mice (Table 3). When the parameters above were related to lean body mass, no significant differences were observed between *Fxr* KO and WT mice (data not shown).

**Table 3:** Water and energy intake and uptake at 29–31 weeks of age.

Parameter	WT	*Fxr* KO
Energy intake (kcal/24 h)	15.14±0.44	14.76±0.59
Faecal energy loss (kcal/24 h)	3.29±0.15	3.09±0.25
Energy uptake (kcal/24 h)	11.84±0.29	11.67±0.47
Relative energy intake (kcal/24 h/bw)	0.42±0.02	0.51±0.02**
Relative faecal energy loss (kcal/24 h/bw)	0.09±0.01	0.11±0.01
Relative energy uptake (kcal/24 h/bw)	0.33±0.02	0.40±0.02*
Water consumption (ml/24 h)	2.47±0.14	2.77±0.17
Relative water consumption (ml/24 h/bw)	0.07±0.01	0.10±0.01**

Values are presented as group mean ± SEM. Body weight at assessment for WT mice (n = 8): 36.7±1.6 g, *Fxr* KO mice (n = 8): 29.1±1.3 g. Statistical analysis performed by Student's t-test.

* p<0.05;

** p<0.01 *Fxr* KO vs. WT mice.

bw; body weight.

**Figure 3:**
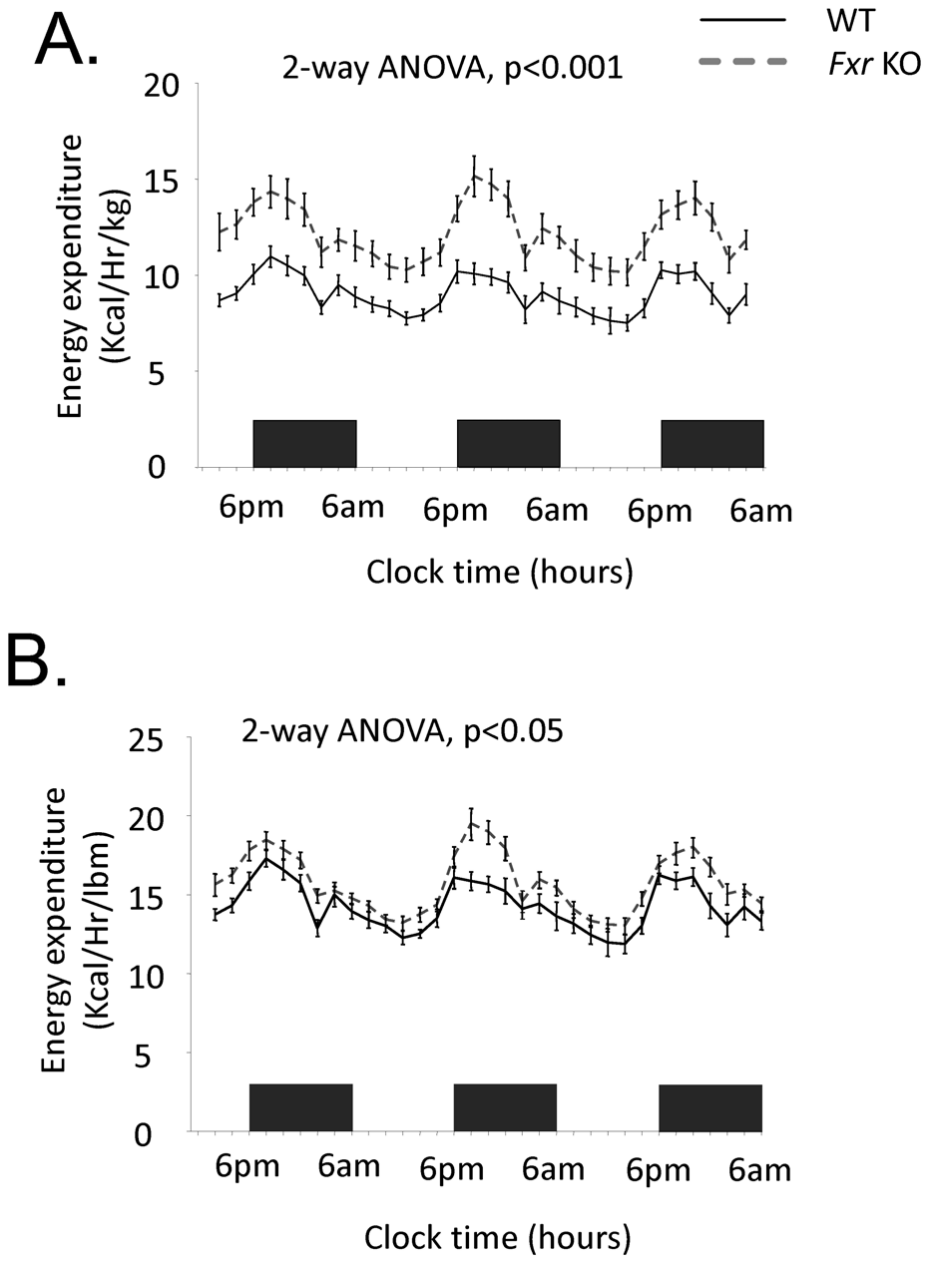
Indirect calorimetry assessment. (A) Energy expenditure relative to body weight assessed in kilocalories per hour and kilogram body weight (kcal/Hr/kg) and (B) energy expenditure relative to lean body mass (lbm) in WT (*n* = 8, black solid line) and *Fxr* KO mice (*n* = 8, grey dashed line). Black bars at the X-axis represent light off. Statistical analysis was performed using a 2 way ANOVA mixed model.

Total energy expenditure (kcal/hr) was not significantly different, whereas energy expenditure related to body weight (Fig. 3A) and energy expenditure related to lean body mass (Fig. 3B) were significantly increased in the *Fxr* KO compared to the WT mice. No significant difference was observed in RER between *Fxr* KO and WT mice at this age (data not shown). Core body temperature was not significantly different between the experimental and control groups of mice (WT: 36.9±0.2°C; *Fxr* KO: 36.6±0.2°C, *p* NS). Total locomotor activity was not significantly different between *Fxr* KO and WT mice over the 72 hour indirect calorimetry analysis (data not shown).

### Oral glucose tolerance test

**Figure 4:**
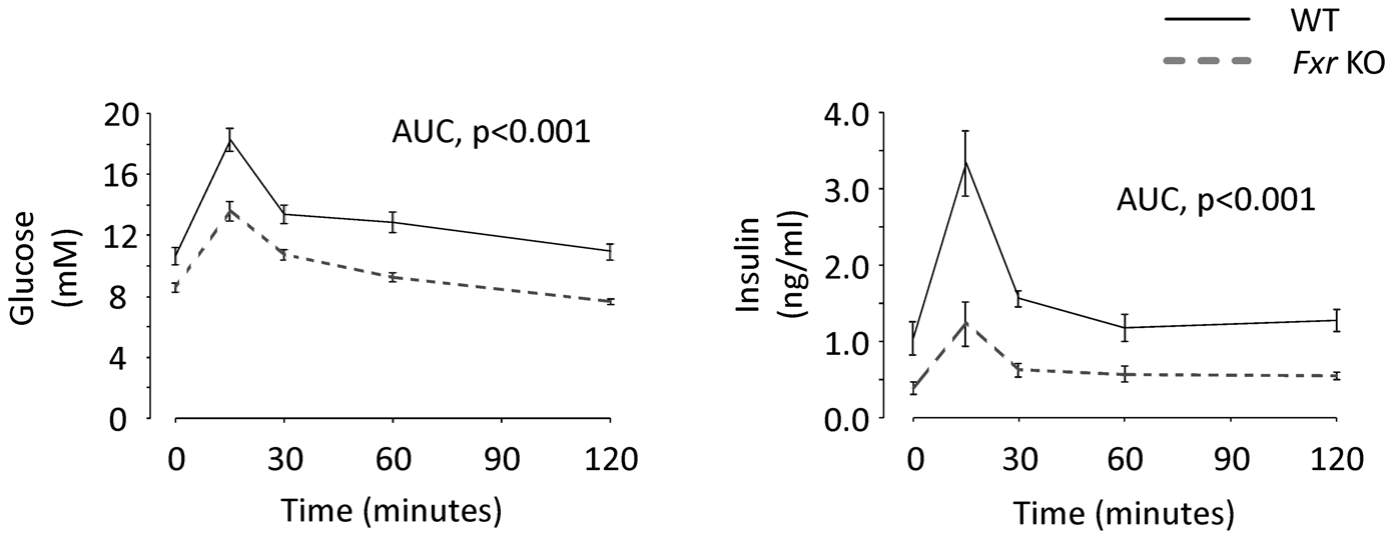
Glucose tolerance. Oral glucose tolerance test in WT (*n* = 7, black solid line) and *Fxr* KO mice (*n* = 7, grey dashed line) mice. Statistical analysis of area under the curve (AUC) values was performed using Student's T-test.

At 30 weeks of age, glucose tolerance was analyzed following 5 hour diet deprivation. The *Fxr* KO mice displayed significantly lower fasting glucose (−19%, *p*<0.01) and fasting insulin (−62% *p*<0.05) levels (Fig. 4) resulting in a 70% lower HOMA-IR index (WT: 0.52±0.15; *Fxr* KO: 0.16±0.03, *p*<0.05). Following an oral glucose challenge (2 g/kg), both the glucose and insulin response were significantly lower in the *Fxr* KO compared to the WT mice (Fig. 4). Glucose AUC was 26% lower (WT: 1568.6±64.4; *Fxr* KO: 1163.5±27.3, p<0.001) and insulin AUC was 58% lower (WT: 184.4±20.2; *Fxr* KO: 78.2±11.5, p<0.001) in the *Fxr* KO compared to the WT mice.

### Tissue weights and hepatic triglyceride content

**Figure 5:**
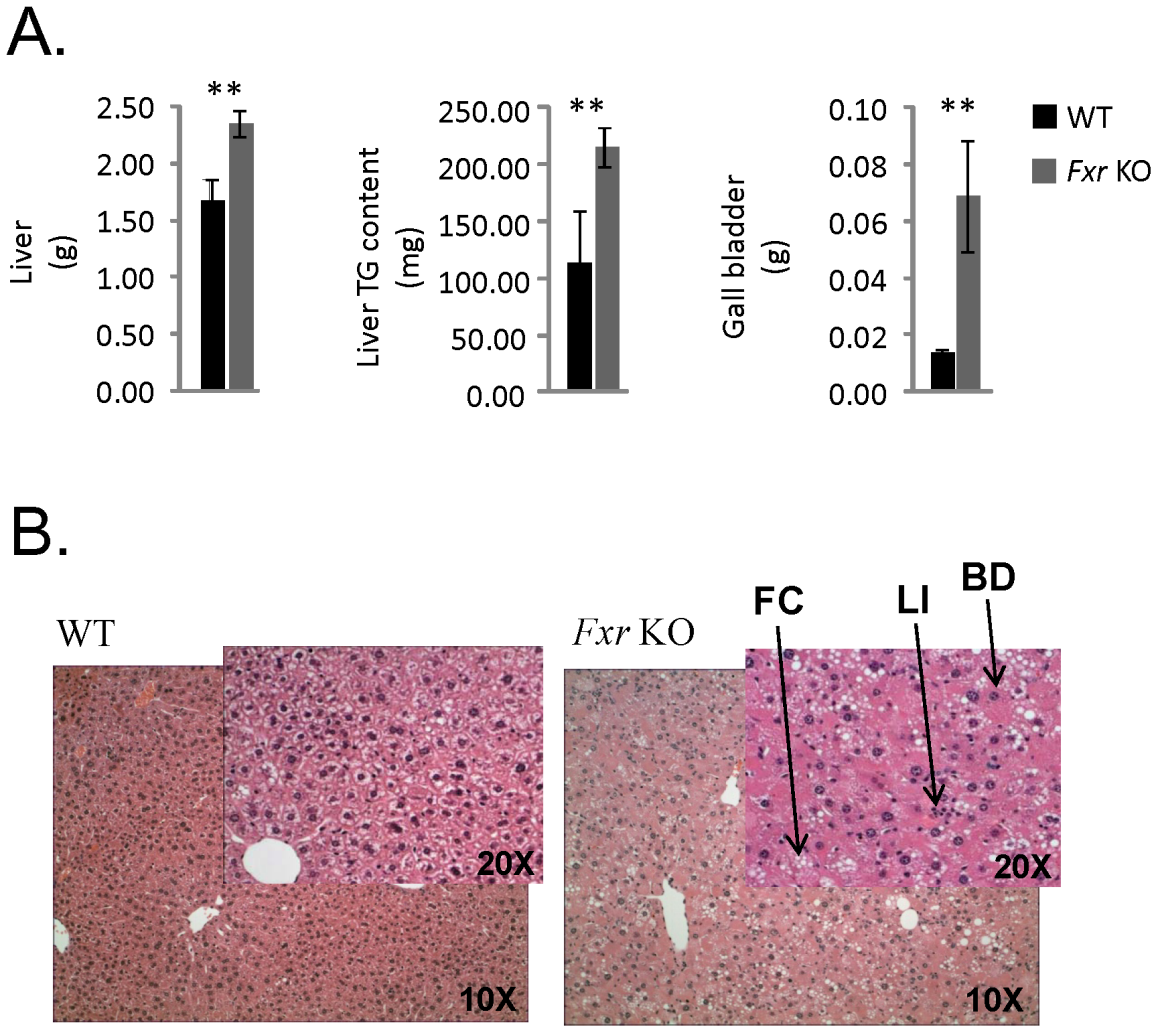
Tissue weight and triglyceride content. (A) Weight of liver, liver triglyceride (TG) content and weight of the gall bladder in WT (*n* = 8, black bars) and *Fxr* KO mice (*n* = 8, grey bars). Statistical analysis was performed using Student's T-test. ** p<0.01 *Fxr* KO vs. WT mice. (B) Representative slides of livers from WT (*n* = 8) and *Fxr* KO mice (*n* = 8) as indicated. The arrows indicate perisinusoidal/sinusoidal foam cells (FC), lobular inflammation (LI) and ballooning degeneration (BD).

The mice were sacrificed at 39 weeks of age and the tissue weights are collected in Table 4. At sacrifice it was noted that the liver, paws and plasma were yellow in colour in the *Fxr* KO mice, indicative of jaundice. The weight of the liver was increased by over 40% (Fig. 5A) and an even greater increase in liver weight was observed when related to individual body weights (WT: 42.5±2.9 mg/g body weight; *Fxr* KO: 79.8±6.6 mg/g body weight, p<0.001). Hepatic levels of triglycerides were significantly increased, both in term of absolute (Fig. 5A) and relative quantities (WT: 55.9±15.7 mg tg/g liver; *Fxr* KO: 91.9±6.8 mg tg/g liver, p<0.05). The non-emptied gall bladder had 5-fold higher weight in the *Fxr* KO compared to the WT mice (Fig. 5A) and relative to body weight the difference between the genotypes was even greater (WT: 0.36±0.04 mg/g body weight; *Fxr* KO: 2.50±0.77 mg/g body weight, p<0.01).

**Table 4:** Absolute and relative (Rel.) tissue weights.

Parameter	WT	*Fxr* KO
**Heart** (g)	0.18±0.01	0.16±0.01
Rel. Heart (mg/g bw)	4.70±0.24	5.33±0.24
**Epi WAT** (g)	1.52±0.27	0.68±0.19*
Rel. epi WAT (mg/g bw)	39.69±5.94	20.91±4.79*
**Retro WAT** (g)	0.46±0.05	0.19±0.06**
Rel. retroWAT (mg/g bw)	11.86±1.06	5.59±1.63**
**BAT** (g)	0.23±0.03	0.08±0.02**
Rel. BAT (mg/g bw)	5.77±0.52	2.73±0.43***
**Testis** (g)	0.22±0.01	0.20±0.01*
Rel. Testis (mg/g bw)	5.77±0.27	6.72±0.31*
**Spleen** (g)	0.07±0.00	0.10±0.01***
Rel. Spleen (mg/g bw)	1.89±0.10	3.52±0.24***
**Kidney** (g)	0.44±0.01	0.35±0.02**
Rel. Kidney (mg/g bw)	11.65±0.67	11.71±0.27
**Caecum** (g)	0.52±0.03	0.59±0.04
Rel. Caecum (mg/g bw)	13.71±.71	20.25±2.05**
**Brain** (g)	0.48±0.00	0.48±0.00
Rel. Brain (mg/g bw)	12.57±0.58	16.06±0.80**

Values are presented as group mean ± SEM. WT mice n = 8, *Fxr* KO mice n = 8. Statistical analysis performed by Student's t-test.

* p<0.05;

** p<0.01;

*** p<0.001 *Fxr* KO vs. WT mice.

WAT; white adipose tissue, Epi; Epididymal, Retro; retroperitoneal, BAT; brown adipose tissue, bw; body weight, Rel.; relative.

Both absolute and relative (relative to body weight) weights of epididymal WAT, retroperitoneal WAT and interscapular BAT was significantly lower, whereas the weight of the spleen and caecum were significantly higher in the *Fxr* KO compared to the WT mice.

### Plasma analysis

All data from the plasma analysis is collected in Table 5. At termination, fasting blood glucose levels were significantly lower in the *Fxr* KO compared to the WT mice. Plasma lipid analysis revealed significantly lower levels of non-esterified fatty acids (NEFA) and higher levels of total cholesterol and non-HDL cholesterol in the *Fxr* KO compared to the WT mice. Plasma levels of total bile acids were more than 30-fold higher in the *Fxr* KO compared to WT mice. Moreover, plasma levels of total bilirubin and alanine aminotransferase (ALT) were more than 20-fold and 4-fold higher respectively in the *Fxr* KO mice, indicating markedly deranged liver function.

**Table 5:** Plasma analyses.

Parameter	WT	*Fxr* KO
Fasting glucose (mM)	10.58±0.60	8.89±0.28*
Total NEFA (mM)	0.42±0.02	0.28±0.03**
Total TG (mM)	0.57±0.06	0.64±0.04
Total Cholesterol (mM)	2.68±0.16	4.14±0.28**
Non HDL cholesterol (mM)	0.40±0.04	2.00±0.27***
HDL (mM)	2.28±0.13	2.14±0.50
Total bilirubin (μM)	2.33±0.38	46.69±17.53*
ALT (μkat/l)	1.13±0.58	5.17±0.70***
Total BA (μM)	10.01±7.90	345.25±122.59**

Values are presented as group mean ± SEM. WT mice n = 8, *Fxr* KO mice n = 8. Statistical analysis performed by Student's t-test.

* p<0.05;

** p<0.01;

*** p<0.001 *Fxr* KO vs. WT mice.

NEFA; Non-Esterified Fatty Acids, ALT; alanine aminotransferase, BA; bile acids.

### Histology and liver expression analyses

**Figure 6:**
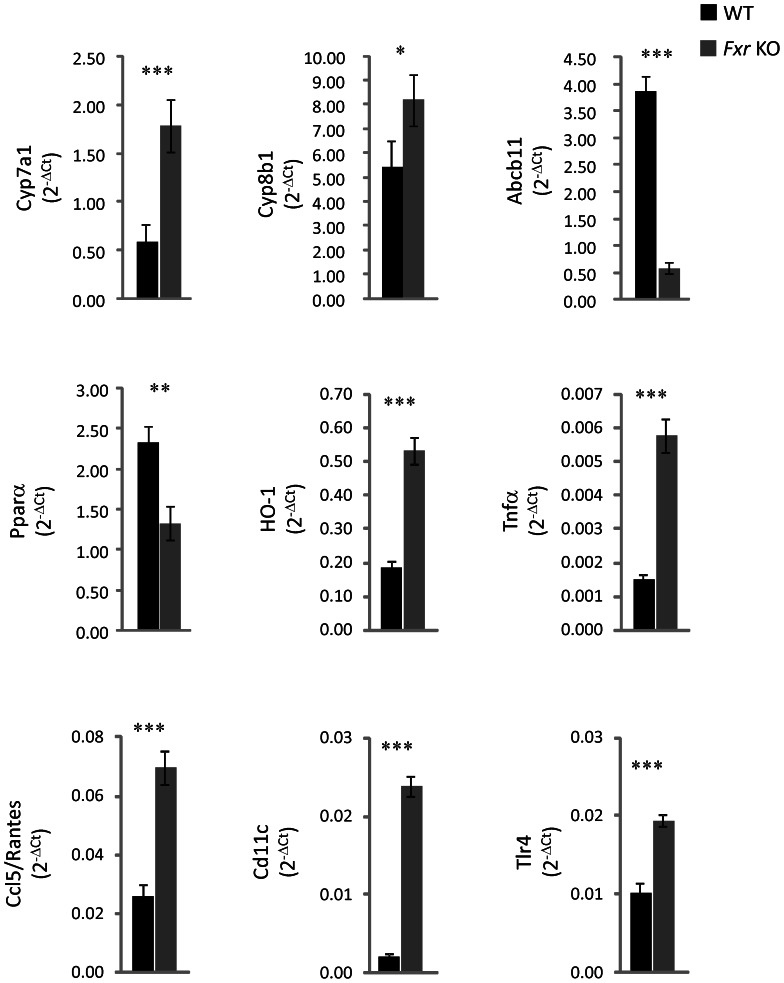
Liver gene expression levels. RNA expression levels in WT (*n* = 8, black bars) and *Fxr* KO mice (*n* = 8, grey bars). Statistical analysis was performed using Student's T-test. * p<0.05, ** p<0.01, *** p<0.001 *Fxr* KO vs. WT mice.

Inferior lobes of the liver were sampled to assess liver histopathology. Livers from the *Fxr* KO displayed higher degree of steatosis, perisinusoidal/sinusoidal foam cells, ballooning degeneration and lobular inflammation compared to WT mice, indicative of liver dysfunction and NASH-like steatohepatitis in the *Fxr* KO mice (Fig 5B). To investigate possible mechanisms behind changes in liver metabolism of bile acids and lipids as well as liver inflammation, expression levels of a series of genes were assessed (Fig. 6). The *Fxr* KO mice displayed significantly increased hepatic expression of Cyp7a1 and Cyp8b1, but markedly lower expression of Abcb11 (bile acid export pump (BSEP)) (Fig. 6). PPARα and two down-stream target genes (medium-chain acyl-CoA dehydrogenase and acyl-CoA oxidase) were analysed to understand if increased PPARα signalling could help to explain the increased energy expenditure, liver size and triglyceride content [32], [33]. Expression level of Pparα mRNA was decreased (Fig. 6) whereas expression of medium-chain acyl-CoA dehydrogenase and acyl-CoA oxidase mRNA was not significantly different between the *Fxr* KO and WT animals (data not shown). Since liver triglyceride levels were increased, while plasma levels of fatty acids were reduced in *Fxr* KO mice as compared to WT controls, key genes involved in *de novo* fatty acid synthesis were analysed. Srebp-1c mRNA expression was lower in *Fxr* KO, while Fas and Scd-1 mRNA expression was not significantly different between the two groups of animals (data not shown). Heme oxygenase -1 (HO-1) mRNA expression was increased more than 2-fold in the *Fxr* KO livers indicating increased oxidative stress in the *Fxr* deficient livers. Tnfα, Ccl5/Rantes, Tlr-4 and Cd11c expression was higher in livers from *Fxr* KO mice than in WT mice indicating severe inflammatory changes (Fig. 6).

**Figure 7:**
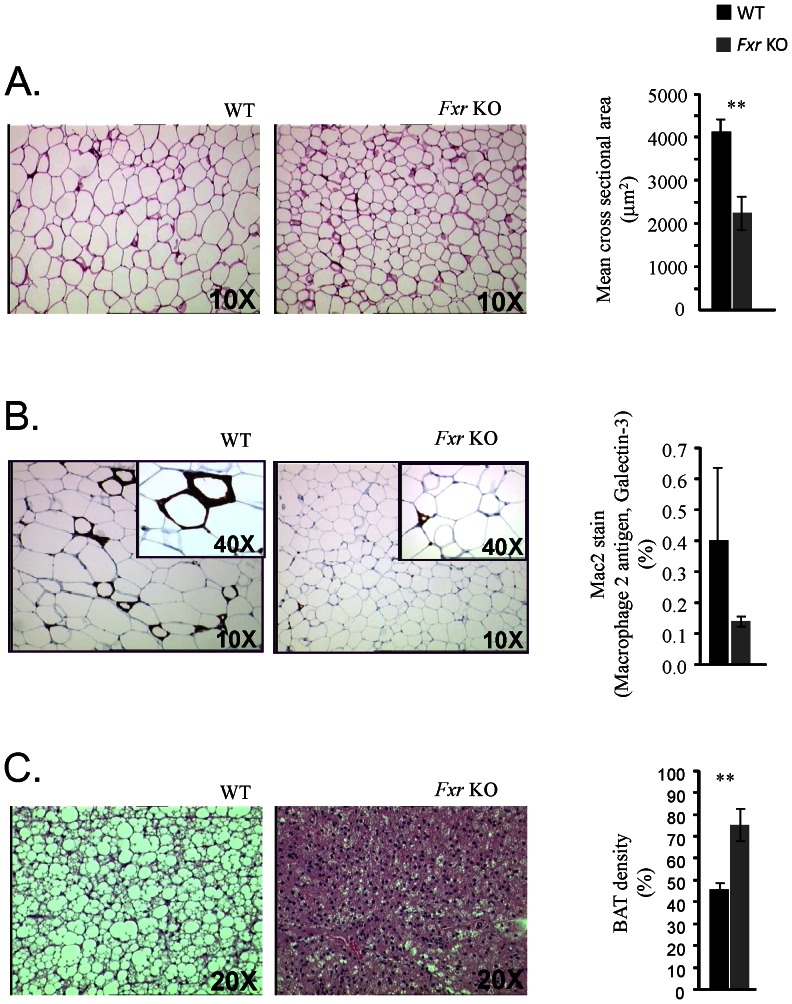
Adipose tissue cell size, inflammation and density assessment. (A) Representative slides of basic fuchsin stained WAT from WT (*n* = 8) and *Fxr* KO mice (*n* = 8) as indicated. (B) Representative slides of WAT stained for Mac2 (Macrophage 2 antigen, Galectin-3) from WT (*n* = 8) and *Fxr* KO mice (*n* = 8) as indicated. (C) Representative slides of hematoxylin- eosin stained BAT from WT (*n* = 8) and *Fxr* KO mice (*n* = 8) as indicated. Statistical analysis was performed using Student's T-test. ** p<0.01 *Fxr* KO vs. WT mice.

**Figure 8:**
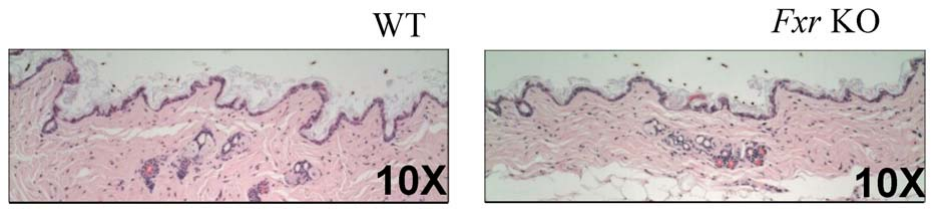
Skin histology. Representative slides of skin biopsies from WT (*n* = 8) and *Fxr* KO mice (*n* = 8) as indicated.

White adipose tissue (WAT) was analysed in terms of cell profile size and macrophage content in sections. Cell profile size distribution analysis of>5000 cells/animal indicated that adipocytes from the *Fxr* KO mice were significantly smaller in size compared to WT mice (Fig. 7A). WAT macrophage content analyses indicated less ‘crown like’ structures in the *Fxr* KO mice, however the quantification did not reveal a statistically significant difference between *Fxr* KO and WT mice (Fig. 7B). The density of the interscapular BAT depot was significantly higher in the *Fxr* KO mice, indicating decreased amount of lipid droplets in BAT from *Fxr* KO compared to WT mice (Fig. 7C). Skin samples were analysed for potential histopathological alterations of epidermis, but no histological skin abnormalities were detected, including no alterations in epidermal thickness or in dermal or epidermal appearance in the *Fxr* KO compared to WT mice (Fig. 8).

## Discussion

The *Fxr* deficient mice from this novel mouse line display reduced body weight gain from 21 weeks of age and onwards which is explained by increased energy expenditure. Moreover, the ageing *Fxr* deficient mice, without previous metabolic challenge, showed a picture of intrahepatic cholestasis and hepatocyte damage resembling non-alcoholic steatohepatitis (NASH). The livers were yellowish, steatotic and enlarged and plasma levels of ALT, bile acids and bilirubin were markedly elevated in the *Fxr* deficient mice. Also, the *Fxr* deficient mice were hypercholesterolemic, possibly as a consequence of the cholestasis. Towards the end of the study, the *Fxr* deficient mice displayed improved glucose tolerance and lower HOMA-IR index indicating improved insulin sensitivity.

In contrast to our results, it has been shown that *Fxr* deficient mice have reduced glucose tolerance and insulin resistance at 8–12 weeks of age [4] and 18–20 weeks of age [7]. Body weight gain was shown to be similar in *Fxr* deficient and WT mice up to 18–20 weeks of age, whereas body fat mass were reduced. The reduced body fat mass was explained by reduced adipocyte differentiation, and it was suggested that an increased flux of fatty acids to skeletal muscle reduced glucose tolerance and insulin sensitivity [7]. In contrast, ageing *Fxr* deficient mice fed a chow diet in the present study displayed reduced body fat mass and lower free fatty acids levels, indicating reduced flux of fatty acids from the adipose tissue. A later study investigated the effect of *Fxr* deficiency in mice fed high fat diet and on a leptin deficient genetic background [22]. In line with the present study, they found reduced body weight gain, body fat mass and smaller adipocytes. Moreover, they showed that *Fxr* deficiency improved glucose tolerance and insulin sensitivity in spite of increased liver fat [22]. Together with our results it is therefore likely that the improved glucose control in older *Fxr* deficient mice independent of genetic background is explained by lower body weight gain and fat mass. In contrast to our findings of increased energy expenditure as an explanation to the reduced body weight gain, the *Fxr* deficient mice on the leptin deficient background displayed reduced energy expenditure, and a trend towards decreased food intake [22]. We extended these previous studies by showing that improved glucose control as a result of *Fxr* deficiency does not only occur after diet induced or genetically induced obesity, but also in association with ageing. It was concluded by Prawitt *et.al*. [22] that the increased plasma bile acid levels could not explain the improved glucose control, since lowering of bile acids using colesevelam did not change glucose levels in *Fxr* deficient animals. However, they observed that the body weight gain was slightly increased by colesevelam, indicating that bile acids could take part in the reduced body weight gain in *Fxr* deficient mice [22]. It is therefore possible that increased plasma bile acid levels in older *Fxr* deficient mice are partly responsible for the reduced body weight gain and increased energy expenditure. Bile acids increase energy expenditure by activating the G-protein-coupled receptor TGR5 in brown adipose tissue and skeletal muscle [34]. It is therefore likely that the increased energy expenditure is at least partly explained by increased bile acid levels activating TGR5. Increased energy expenditure is a powerful way of enhancing glucose control via increased insulin sensitivity as exemplified by brown adipose tissue transplantation [35], increased energy expenditure in the context of increased inflammation [36] or a primary defect in the skin resulting in increased heat loss and energy expenditure [37]. The most plausible explanation to the improved glucose tolerance in the ageing *Fxr* deficient mice is therefore increased energy expenditure.

The ageing *Fxr* deficient mice did not gain body weight and had a markedly reduced percentage of body fat as compared to the WT mice. Since there were no significant differences in energy intake or fecal energy loss, the reduced body weight gain is most likely explained by the increased energy expenditure. There are several potential explanations to the increased energy expenditure in the *Fxr* deficient mice and some of these explanations were ruled out in this study. *Fxr* deficiency was not accompanied by increased locomotor activity or core body temperature and the unaltered body temperature together with the lower weight of the BAT depot argues against a primary effect in that tissue. However, we cannot rule out that TGR5 activation of energy production in BAT and skeletal muscle contributed to the increased energy production. Thus, increased energy expenditure that is not explained by increased locomotor activity or core body temperature could possibly be explained by increased heat loss via the skin. Although no major structural changes of the skin were observed on the *Fxr* deficient mice, it does not exclude that the increased energy expenditure in older *Fxr* deficient animals is explained by increased heat loss through superficial vasodilatation and activation of counter-current temperature systems in skin, nasal conchae and tail. Hypothetically, direct epidermal convection heat loss cannot be excluded, and would possibly be supported by the finding that FXR may be of importance for development of the fetal epidermal permeability barrier [38]. The increased weight of the liver observed in the present study in the *Fxr* deficient mice together with previous findings suggesting that PPARαactivation can contribute to increased energy expenditure [33] led us to investigate whether there are signs of increased PPARα activation in the liver from the *Fxr* deficient mice. Liver expression of Pparα was reduced but there were no changes in two down-stream target genes indicating no major change in Pparα signalling. We therefore believe it is unlikely that increased PPARα activation could have contributed to increased energy expenditure and liver weight. The increased liver weight was not explained by the increased liver triglyceride content since it increased from approximately 6% in WT controls to 9% of the total liver weight in the *Fxr* deficient mice. Increased liver triglycerides in *Fxr* deficient mice have been reported before [7], [24]. Increased flux of fatty acids to the liver because of reduced adipogenesis [7] as well as increased hepatic *de novo* lipogenesis has been suggested to explain increased liver triglyceride content in the *Fxr* deficient mice [4], [24], [39]. In the present study, we did not find evidence for either increased *de nov*o lipogenesis or increased flux of fatty acids. It is therefore most likely that the small increase in liver triglycerides is a sign of hepatocyte dysfunction resulting in retention of lipids. In line with our findings, *Fxr* deficient mice at 15 months of age display increased liver size [16]. Increased liver expression of cyclin D1 and E1 of old *Fxr* deficient mice indicated that the cause of the increased liver size was increased cell division. Hence, the increased liver weight in the *Fxr* deficient mice is likely explained by hyperplasia.

The *Fxr* deficient mice suffered from cholestasis as indicated by yellowish discoloured liver, paws and plasma and very high plasma levels of bilirubin and bile acids. As expected from previous studies [5], [39], the expression of Cyp7a1 and Cyp8b1 were markedly increased in the *Fxr* deficient mice, most likely resulting in increased production of bile acids. Normal expression of *Abcb11* (BSEP) is rate-limiting for pumping bile acids from the hepatocytes into the bile canaliculi. The expression of *Abcb11* was markedly reduced in the *Fxr* deficient mice as shown before [39] and reduced function or expression of *Abcb11* results in a clinical syndrome of intrahepatic cholestasis [40], [41]. Interestingly, hepatic overexpression of *Abcb11* reduced hepatic lipids [42], indicating that the markedly reduced *Abcb11* expression could contribute not only to cholestasis but also to the observed increase in liver triglycerides.

The old *Fxr* deficient mice in the current study displayed a picture of cholestasis and steatohepatitis despite improved glucose control and no dietary challenges. Very high plasma levels of bile acids, bilirubin, ALT and cholesterol indicated dysfunctional liver metabolism and hepatic damage in the *Fxr* deficient mice. The histological examination revealed high degree of perisinusoidal/sinusoidal foam cells, ballooning degeneration and lobular inflammation in the *Fxr* deficient animals. A similar pathology has been described to occur in livers of *Fxr* deficient mice on *Ldlr* deficient background, and more pronounced when fed HFD [24]. The liver of the old *Fxr* deficient mice had increased expression of Tnfα, HO-1, Ccl5, Tlr-4 and Cd11c. Increased expression of TNFα is a common feature of different models of NASH [43] and observed in other *Fxr* deficient models displaying liver injury [16], [17], [24]. Increased oxidative stress also seems to be a common feature of various models of NASH [43]. NASH patients have increased HO-1 expression and the increased expression is regarded as an adaptive response protecting against oxidative damage [44]. Marked induction of HO-1 expression following BACH1 deficiency protects from methionine-choline deficiency induced NASH [45]. Ccl5 mRNA expression was increased more than 2-fold. CCL5 is a chemokine with potent chemoattractant activity for T-lymphocytes and monocytes that have been shown to promote hepatic inflammation and fibrosis in a bile duct ligation model. Moreover, patients with cirrhosis show marked up-regulation of Ccl5 mRNA [46]. In line with increased Ccl5 expression, the expression of Cd11c was increased indicating increased myeloid cell activity or number.

A remaining question is what mechanisms could contribute to inflammatory changes resulting in steatohepatitis in ageing *Fxr* deficient mice. One possibility is indicated from studies of the antibacterial defence in the small intestine of *Fxr* deficient animals [47]. It was shown that *Fxr* deficient mice display increased levels of ileal bacteria and a reduced gut barrier function. In the current study, *Fxr* deficient mice displayed markedly increased weight of the ceacum, indicating changed gut microbiota. Thus, it is possible that increased amounts of bacterial products, e.g. endotoxins, might have contributed to the liver damage and inflammation observed in the ageing *Fxr* deficient mice. Indeed, *Tlr-4* deficiency has been shown to protect from diet induced liver inflammation and injury [48] and the present results showing increased expression of the Tlr-4 receptor indicates increased susceptibility to a second hit involving endotoxins. Moreover, it has been suggested that FXR is important for liver repair by promoting regeneration and preventing cell death [17]. Hence, the effects observed in the present study of liver dysfunction may partly be explained by the lack of the liver repair functions of FXR in combination with the cholestasis.

An interesting observation in the present study is that ageing *Fxr* deficient mice had reduced bone mineral density and content. Few studies have investigated the importance of FXR for bone metabolism [49]. They found that FXR activation of human bone marrow stromal cells differentiate these cells into osteoblasts. In contrast, FXR inhibition resulted in an adipocytes-like phenotype [49]. Therefore, it is likely that the ageing *Fxr* deficient mice experience reduced bone mass because of reduced osteoblast differentiation and function.

In summary, this study extends previous observations about the importance of *Fxr* in metabolic regulation by studying single housed male mice of a novel *Fxr* deficient mouse line during ageing. The study sheds light on the long-term consequences of *Fxr* deficiency under normal dietary conditions on energy-, glucose and lipid metabolism. The most striking observation was the serious liver damage including cholestasis and steatohepatitis, resembling NASH, which occurred in the context of leanness and improved glucose homeostasis, strengthening the view that FXR is of major importance for normal liver functions. We conclude that FXR antagonism does not represent an attractive drug target.
